# Apparent Metabolizable Energy and Amino Acid Digestibility of Corn of Different Origin Fed to Male Broilers from 12 to 18 Days of Age

**DOI:** 10.3390/ani13193111

**Published:** 2023-10-06

**Authors:** Jose I. Vargas, Joseph P. Gulizia, Susan M. Bonilla, Santiago Sasia, Wilmer J. Pacheco

**Affiliations:** Department of Poultry Science, Auburn University, Auburn, AL 36849, USA

**Keywords:** amino acid digestibility, apparent metabolizable energy, broilers, corn origin

## Abstract

**Simple Summary:**

Understanding differences in the utilization of corn nutrients by broilers is essential for poultry nutritionists and feed producers that use corn of different origin in their feed formulation programs. In the present study, broilers were fed diets formulated with corn samples from the United States, Argentina, and Brazil to assess apparent metabolizable energy and amino acid digestibility. The results indicated similar nutrient utilization by broilers, regardless of the origin.

**Abstract:**

Apparent metabolizable energy (AME) and apparent ileal amino acid digestibility (AIAAD) of corn samples from the United States (USA), Argentina (ARG), and Brazil (BRA) fed to 432 and 324 YPM × Ross 708 male broilers from 12 to 18 d of age were evaluated using the total collection method (experiment 1) and index method (experiment 2), respectively. In experiment 1, broilers were fed either a basal diet with 30% dextrose inclusion, or a test diet obtained by the replacement of dextrose with corn from each origin. In experiment 2, broilers were fed one of three test diets in which corn from each origin was the only source of AA. All dietary treatments had 12 replicate cages. Similar (*p* > 0.05) AME (dry-matter basis) values were observed between corn samples. The highest gap in AME (43 kcal/kg) was found between corn from BRA (3694 kcal/kg) and corn from the USA (3651 kcal/kg). Likewise, similar (*p* > 0.05) AIAAD values were observed for all AA apart from tryptophan (Trp), as corn from BRA (77.45%) had a higher (*p* = 0.024) Trp digestibility than corn from the USA (72.53%). Overall, a similar nutrient utilization by the birds was observed for the corn samples, regardless of origin.

## 1. Introduction

Corn is an important feed ingredient of broiler diets due to its contribution of energy [1,2,3] and amino acids (AA) [4,5]. In fact, in a typical broiler diet, corn can contribute approximately 65% of apparent metabolizable energy (AME) and 20% of crude protein (CP) [1]. Although corn is considered a consistent ingredient, its nutritional value can vary considerably [1,6], which can impact the economic revenue of broiler production [6]. Genetics [7], agronomic conditions [4], drying conditions [8,9,10,11], and storage [12] can influence its nutritional value.

Variability in the AME of corn can be associated with differences in the content of starch (67.8–76.1% dry matter (DM)) [13], digestibility of starch [14,15], oil concentration (44–123 g/kg DM) [5], fiber content (14.3–17.1% DM) [13], and non-starch polysaccharides concentration (9 ± 9% DM) [16]. Variability in AA digestibility of corn can be associated with differences in the content of CP (78–112 g/kg DM) and concentration of AA (Methionine (Met): 1.6–2.6 g/kg DM; Lysine (Lys): 2.4–3.5 g/kg DM) [5], content of phytate [17], and drying temperatures [18,19,20]. The determination of AME and apparent ileal AA digestibility (AIAAD) of corn from different countries is important during feed formulation. However, there is limited scientific literature evaluating differences in AME and AIAAD of corn from different origin. Therefore, the objective of the study was to determine the AME (experiment 1) and AIAAD (experiment 2) of corn from the United States (USA), Argentina (ARG), and Brazil (BRA) fed to male broilers from 12 to 18 d of age.

## 2. Materials and Methods

### 2.1. Animal Care

The poultry experiments reported herein were reviewed and approved by the Auburn University Institutional Care and Use Committee (PRN 2021-3874).

### 2.2. Bird Husbandry

A total of 432 and 324 broiler chickens were obtained from a parent stock flock YPM × Ross 708 (Aviagen North America, Huntsville, AL, USA) and randomly sorted into 48 and 36 battery cages (9 birds/cage; 0.05 m^2^/bird) (Petersime, Gettysburg, OH, USA) for the AME and AIAAD experiment, respectively.

Both experiments were conducted simultaneously in a solid-sided experimental facility with electronic temperature control. Each cage had 1 trough feeder and 1 trough waterer. The temperature was set at 33 °C at placement and gradually decreased to 26.1 °C upon conclusion of the trial. A 23L:1D lighting regime with an intensity of 4.0-foot candles was used for the whole trial. Feed and water were provided ad libitum throughout the experimental period. All broilers were subjected to a common corn–soybean meal-based starter diet until 11 d of age and the experimental dietary treatments from 12 to 18 d of age. The common starter and all dietary treatments were provided in mash form.

### 2.3. Feed Formulation, Manufacture, and Experimental Design

Whole corn from the USA (re-imported), ARG, and BRA was ground and analyzed for its physical and nutritional characteristics. The grinding methodology as well as the analyses methodology and results were described in detail by Vargas et al. [21]. This was a previous project from our research group in which the effect of the dietary inclusion of the same corn samples was evaluated on broiler performance. An extract of the analyzed nutrient composition results is presented in Table 1.

The percentage of broken corn and foreign material (BCFM) and test weight (kg/hL) for the corn samples were 8.20, 1.30, and 2.20% and 72.63, 72.50, and 73.13 kg/hL for corn from the USA, ARG, and BRA, respectively [21].

All dietary treatments were produced at the Auburn University Poultry and Animal Nutrition Center. Ingredients were mixed for 150 s (30 s dry cycle and 120 s wet cycle) using a twin shaft mixer (Model 726, Scott Equipment Co., New Prague, MN, USA) to produce the mash diets.

In experiment 1, dietary treatments consisted of a grower corn–soybean meal-based basal diet with 30% dextrose inclusion, and 3 test diets obtained by the substitution of dextrose from the basal diet with corn from different origins: USA, ARG, and BRA (Table 2). Each cage was randomly assigned to 1 of 4 experimental diets, obtaining 12 replicates per treatment.

In experiment 2, dietary treatments consisted of 3 test diets with corn from the USA, ARG, and BRA being the only source of AA (Table 3). Diets were fortified with minerals and vitamins, and dextrose was provided as a source of energy. An indigestible marker (0.5% titanium dioxide (TiO_2_)) was added to the test diets for AIAAD calculations. Each cage was randomly assigned to 1 of the 3 experimental diets, obtaining 12 replicates per treatment.

### 2.4. Metabolizable Energy Assay

In experiment 1, birds were fed the experimental diets from 12 to 18 d of age, the period in which feed intake (FI) and body weight (BW) were monitored to ensure the acceptance of the dietary treatments. Starting at 16 d of age, a 72 h energy balance assay using the total collection method was conducted until 18 d of age. Feed intake and excreta weights (wet-basis) were recorded during the 72 h collection period to calculate energy intake and excretion. For each cage, a 250 g excreta representative sample (free from feathers and feed) was obtained on each day of the assay. Samples were collected from the accumulated excreta on the pan placed beneath each cage, stored in a resealable plastic bag, and transported to the laboratory for further processing. A 40 g representative subsample was obtained from each bag, transferred to a cup, and frozen at −20 °C. Excreta samples were freeze-dried in a Virtis Genesis Lyophilizer (SP Industries, Warminister, PA, USA), followed by grinding in an electric coffee grinder (Capresso 560.4 Infinity, Montvale, NJ, USA). After grinding, equal aliquots of samples from different days were pooled by cage for gross energy (GE) analysis. Gross energy of the feed and excreta samples were analyzed in duplicate using a sample of 0.8 g by the Central Analytical Laboratory of the University of Arkansas using an adiabatic oxygen bomb calorimeter (Parr Instruments, Moline, IA, USA).

Total excreta weight, FI, and GE data were introduced in the following equation for the calculation of metabolizable energy coefficient (MEC):

$$MEC,\;(\%)=\left\{\frac{\left[FI\times {Energy}_{feed}\right]-\left[Excreta\;output\times {Energy}_{excreta}\right]}{FI\times {Energy}_{feed}}\times 100\right\}$$

where FI represents feed intake (kg), ${Energy}_{feed}$ and ${Energy}_{excreta}$ represent the GE (kcal/kg) of the experimental feeds and excreta, respectively, and Excreta output represents the weight (kg) of the excreta collected during the same period as FI.

The AME of the basal and test diets was calculated using the following formula:

$${AME}_{\;basal\;and\;test\;diets},\left(\frac{\mathrm{kcal}}{\mathrm{kg}}\right)=\frac{MEC\times {Energy}_{\;diet}}{100}$$

where ${AME}_{basal\;and\;test\;diets}$ represents AME of the basal and test diets, MEC represents the metabolizable energy coefficient, and ${Energy}_{diet}$ represents the GE (kcal/kg) of basal diet and test diets.

The AME of the test ingredient (corn from different origin) was calculated with the standard ingredient substitution method described recently by Wu et al. [22], in which an ingredient with known AME such as dextrose (3640 kcal/kg) [23,24] is used to prepare the basal diet, and replaced by the test ingredient to create the test diets.

Finally, the AME of the corn from different origin was calculated by the following formula adapted from Wu et al. [22]:

$${AME}_{\;corn},\left(\frac{\mathrm{kcal}}{\mathrm{kg}}\right)={AME}_{dextrose}-\left({AME}_{test\;diet}-{AME}_{basal\;diet}\right)$$

where ${AME}_{\;corn}$ represents AME of the corn samples from different origin, ${AME}_{dextrose}$ represents AME of dextrose, ${AME}_{test\;diet}$ represents the AME of the test diet, and ${AME}_{basal\;diet}$ representes the AME of the basal diet. All presented calculations were performed on a DM basis.

### 2.5. Amino Acid Digestibility Assay

In experiment 2, birds were fed the experimental diets from 12 to 18 d of age to measure AIAAD using the index method. At 18 d of age, all birds from each cage were euthanized by CO_2_ asphyxiation, followed by cervical dislocation performed by trained personnel. Ileal digesta was collected from 2 cm posterior of Meckel’s diverticulum to the 2 cm anterior of the ileal–cecal junction by gently flushing the ileum with distilled, deionized water, and squeezing out the ileal contents into a cup. The ileal contents were pooled by pen and kept on ice until transported to the laboratory. Samples were frozen at −20 °C and freeze-dried in a Virtis Genesis Lyophilizer (SP Industries, Warminister, PA, USA), followed by grinding in an electric coffee grinder (Capresso 560.4 Infinity, Montvale, NJ, USA). The concentration of TiO_2_ was determined in duplicate for ground digesta and in triplicate for dried diets, following the procedures of Short et al. [25].

Absorbance was measured using a spectrophotometer (Shimadzu, Kyoto, Japan) using 1 mL of the sample, and a standard curve was used to calculate TiO_2_ concentration of the digesta and feed samples. Digesta and feed samples were analyzed for AA profile using HPLC (method 982.30 E (a,b,c), chp. 45.3.05 and method 988.15, chp. 45.4.04; AOAC International, [26]) by the Agricultural Experiment Station Chemical Laboratories of the University of Missouri-Columbia. Apparent ileal AA digestibility was calculated using the following equation, adapted from Kong and Adeola [27]:

$$AIAAD,\;(\%)=\left[1-\left(\frac{{Ti}_{diet}}{{Ti}_{digesta}}\right)\times \left(\frac{{AA}_{digesta}}{{AA}_{diet}}\right)\right]\times 100$$

where ${Ti}_{diet}$ and ${Ti}_{digesta}$ represent the concentration of TiO_2_ in diet and digesta, respectively, and ${AA}_{digesta}$ and ${AA}_{diet}$ represent the concentration of the studied AA in digesta and in diet, respectively. The use of this formula assumed that AIAAD of the test diet is representative of the corn samples of different origin, since they were the only dietary source of AA for the birds within the test diets.

### 2.6. Statistical Analyses

All data were analyzed as a randomized complete block design, with cage location considered as the blocking factor. Each treatment had 12 replications, with cage being the experimental unit. Data were analyzed as a one-way ANOVA using the GLM procedure of JMP software [28]. The least square means among the treatments were compared using Tukey’s HSD procedure with statistical significance considered at *p* ≤ 0.05.

## 3. Results

### 3.1. Apparent Metabolizable Energy

Apparent metabolizable energy results can be observed in Table 4. No statistical differences (*p* > 0.05) were observed in AME among the corn samples from different origin. Numerically, corn from BRA (3694 kcal/kg DM) had the highest AME, followed by samples from ARG (3666 kcal/kg DM) and the USA (3651 kcal/kg DM). Percentages of absorptions of GE by the birds of corn samples from BRA, ARG, and the USA were 84.04, 82.90, and 82.66%, respectively. Apparent metabolizable energy of the basal diet used for the standard ingredient substitution method was 3229 kcal/kg DM.

### 3.2. Apparent Ileal Amino Acid Digestibility

Apparent ileal amino acid digestibility results can be observed in Table 5. No statistical differences (*p* > 0.05) were observed on AIAAD among corn from different origin for all analyzed AA, but Trp. Broilers fed corn from BRA (77.45%) had higher (*p* = 0.024) Trp digestibility than broilers fed corn from the USA (72.53%), but not different (*p* > 0.05) than corn from ARG (73.30%).

The mean digestibility of the corn samples for Lys, Met, and Thr was 69, 85, and 66%, respectively. Among all AA, the highest mean digestibility was calculated for Leu (88%) and the lowest for Thr (66%). The highest difference on AIAAD was found for Trp between corn from BRA and corn from the USA at 4.92%.

## 4. Discussion

The AME results of the present report are in close agreement with the findings by Labiski and Anderson [29] and Barzegar et al. [30] who calculated the % of utilization of GE of corn by birds at 84.8 and 85.0%, respectively. In experiment 1, AME increased as the oil content of the corn samples increased; corn from BRA had the highest AME and content of oil, followed by corn from ARG, and corn from the USA. Other researchers have evaluated the impact of oil content on metabolizable energy [31,32,33,34], indicating an increase in metabolizable energy along with an increase in oil content. Recent research by Zuber and Rodehutscord [5] reported a positive correlation between AME corrected to nitrogen equilibrium (AME_n_) and the concentration of oil in corn. In addition, it is possible that a higher content of oil might have contributed to a higher digestibility of dietary ingredients by increasing gastrointestinal retention time, leading to better digestion [35].

The numerical lower AME of the corn sample from the USA in comparison to corn from ARG and BRA could be attributed to a higher content of BCFM compared to corn from BRA and ARG. Rodrigues et al. [36] observed a higher AME of corn samples with fewer impurities (straw, husk or small grains which pass through a 5 mm sieve). Leeson and Summers [19] quantified reductions in AME_n_ of 200 and 600 kcal/kg for broken kernels and foreign material, respectively, in comparison to whole corn kernels. Additionally, differences in test weight might have also contributed to differences in AME [37,38] as corn from BRA had the highest AME (3694 kcal/kg DM) and highest test weight (73.13 kg/hL). Finally, the slightly higher AME values of South American corn samples in comparison to the AME of the corn sample from the USA, could be attributed to a higher content of amylopectin, characteristic of kernels with an increased hardness [39], observed on flint-endosperm cultivars (Zea mays var. indurata), typically used in South America compared to dent-endosperm cultivars (Zea mays var. indentata), typically used in the USA [40]. In fact, the branched structure of amylopectin offers many sites where enzymatic activity and hydrolysis can take place, which could increase AME [39,41].

Overall, our results are lower than the findings of Zuber and Rodehutscord [5], who determined the AME_n_ of 20 corn samples using 55-week cecectomized laying hens, reporting values ranging from 3845 to 3940 kcal/kg DM. Furthermore, Barzegar et al. [30], calculated the AME of corn at 3791 kcal/kg DM using 42-week Hy-Line Brown hens, and a substitution of 30% of a basal diet with corn. These differences could be attributed to differences in the gastrointestinal tract development of the birds due to differences in age [30,42,43]. A more mature gastrointestinal tract of laying hens in comparison to broiler chickens might have increased their nutrient absorptions capacities, deriving more AME from corn in comparison to broilers [42,44]. The AME values of the present experiment are higher than the results of Lopez and Lesson [43], who reported an AME of corn of 3352 kcal/kg DM using 9 to 12-day old broilers, and a substitution of 25% of a basal diet with corn. Likewise, the values of the present experiment are higher than the values obtained by Gehring et al. [6], who calculated AME_n_ values ranging from 3262 to 3342 kcal/kg DM of eight corn samples from different locations within the USA, using broilers fed diets with 99.5% inclusion of corn from 28 to 30 d of age. Perttila et al. [45] reported an AME of corn of 3678 kcal/kg DM using 24-day old Ross broilers fed a semi-purified diet.

In experiment 2, concentrations of all analyzed AA were within ranges previously reported for corn [46]. The higher concentration of Trp of corn from BRA could be associated with its higher oil content, as reported previously by Singh et al. [47], who found a positive correlation between oil content and Trp concentration. In addition, Zuber and Rodehutscord [5] reported a correlation between the concentration of Trp in corn and its digestibility.

The mean digestibility of corn samples for Lys, Met, and Thr in the present experiment is similar to the findings of Huang et al. [48] who reported apparent ileal digestibility coefficients of corn of 69, 87, and 61% for Lys, Met, and Thr, respectively, using 14-day-old Cobb 500 broilers. Ravindran et al. [46] reported apparent ileal digestibility coefficients for corn of 79, 87, and 68% for Lys, Met, and Thr, respectively, using 35–42-day-old broilers. Nonetheless, lower AA digestibility coefficients in young birds have been associated with an underdevelopment of the gastrointestinal tract that does not allow for complete protein digestion [49]. Huang et al. [48] reported higher (*p* < 0.05) apparent AIAAD values for Lys, Met, Thr, Val, Leu, Ile, Phe, and His of corn by 28 and 42-day-old broilers, in comparison to 14-day-old broilers. Alternatively, the low concentration of AA of corn and consequent low AA intake of birds reduce apparent AA digestibility values by increasing the proportion of endogenous protein in the terminal ileum in comparison to undigested protein of dietary origin [46].

The high digestibility of Leu for all corn samples agrees with the study by Ravindran et al. [46], which reported Leu as the AA with the highest digestibility in corn. In contrast, the low digestibility of Thr for all corn samples could be explained by its high concentration in endogenous secretions [50], which increases the amount of recovered Thr, lowering digestibility coefficients [46]. Finally, the highest difference on digestibility between samples exhibited by Trp, agrees with the report by Zuber and Rodehutscord [5] using cecectomized laying hens, in which Trp digestibility among 20 corn samples showed the highest variability.

## 5. Conclusions

In conclusion, AME and AA digestibility did not significantly vary among corn samples of different origin. This can be attributed to the fact that the differences found in physical characteristics and proximate composition among corn samples were smaller than expected. Apparent metabolizable energy and AIAAD values in the present experiment were consistent with previous research using young birds of similar age.

## Figures and Tables

**Table 1:** Analyzed nutrient composition (% dry matter basis) of corn samples of different origin used for the manufacture of the experimental dietary treatments fed to YPM × Ross 708 male broilers from 12 to 18 d of age (experiment 1 and 2) ^1^.

Nutrient	USA	ARG	BRA
Starch	82.78	81.36	79.64
Crude protein	9.49	9.85	9.45
Oil	4.50	4.76	5.20
**Amino Acids**			
Arginine	0.35	0.33	0.34
Cysteine	0.19	0.18	0.19
Histidine	0.26	0.25	0.25
Isoleucine	0.33	0.33	0.31
Leucine	1.00	1.01	0.92
Lysine	0.28	0.27	0.27
Methionine + cysteine	0.35	0.33	0.35
Methionine	0.16	0.15	0.17
Phenylalanine	0.43	0.43	0.39
Threonine	0.29	0.30	0.29
Tryptophan	0.05	0.05	0.06
Valine	0.42	0.42	0.40

^1^ Adapted from Vargas et al. [21].

**Table 2:** Ingredient and nutrient composition (% as-fed basis, unless otherwise indicated) of grower experimental diets fed to YPM × Ross 708 male broilers from 12 to 18 d of age (experiment 1).

Ingredient	Basal Diet	Test Diet USA	Test Diet ARG	Test Diet BRA
Corn USA	-	30.00	-	-
Corn ARG	-	-	30.00	-
Corn BRA	-	-	-	30.00
Local corn ^1^	27.89	27.89	27.89	27.89
Soybean meal, 48.9% CP	37.87	37.87	37.87	37.87
Dextrose	30.00	-	-	-
Poultry oil	1.48	1.48	1.48	1.48
Limestone	0.98	0.98	0.98	0.98
Dicalcium phosphate, 18% P	0.66	0.66	0.66	0.66
Salt	0.42	0.42	0.42	0.42
DL-Methionine, 99%	0.34	0.34	0.34	0.34
L-Lysine	0.06	0.06	0.06	0.06
L-Threonine, 98%	0.08	0.08	0.08	0.08
Choline chloride, 60%	0.06	0.06	0.06	0.06
Trace mineral premix ^2^	0.10	0.10	0.10	0.10
Vitamin premix ^3^	0.08	0.08	0.08	0.08
OptiPhos^®^ Plus ^4^, g/kg	0.12	0.12	0.12	0.12
**Calculated analysis**				
AME_n_^5^, kcal/kg	3100	-	-	-
Crude protein	20.77	-	-	-
Digestible Lys	1.15	-	-	-
Digestible TSAA ^6^	0.87	-	-	-
Digestible Thr	0.77	-	-	-
Digestible Ile	0.82	-	-	-
Digestible Val	0.87	-	-	-
Digestible Arg	1.27	-	-	-
Calcium	0.87	-	-	-
Available phosphorus	0.44	-	-	-

^1^ Corn routinely used for broiler feed production at the Auburn University Feed Mill (CP: 8.49% DM; Oil: 3.90% DM). ^2^ Mineral premix includes per kg of diet: Mn (manganese sulfate), 120 mg; Zn (zinc sulfate), 100 mg; Fe (iron sulfate monohydrate), 30 mg; Cu (tri-basic Copper chloride), 8 mg; I (ethylenediaminedihydroxide), 1.4 mg; Se (sodium selenite), 0.3 mg. ^3^ Vitamin premix includes per kg of diet: Vitamin A (Vitamin A acetate), 187,390 IU; Vitamin D (cholecalciferol), 6614 IU; Vitamin E (DL-alpha tocopherol acetate), 66 IU; menadione (menadione sodium bisulfate complex), 4 mg; Vitamin B12 (cyanocobalamin), 0.03 mg; folacin (folic acid), 2.6 mg: D-pantothenic acid (calcium pantothenate), 31 mg; riboflavin (riboflavin), 22 mg; niacin (niacinamide), 88 mg; thiamin (thiamin mononitrate), 5.5 mg; D-biotin (biotin), 0.18 mg; and pyridoxine (pyridoxine hydrochloride), 7.7 mg. ^4^ OptiPhos^®^ Plus (Huvepharma Inc., Peachtree City, GA, USA) provided 1000 FTU/kg of phytase activity per kg of diet. ^5^ Apparent metabolizable energy corrected to nitrogen equilibrium. ^6^ Total sulfur amino acids.

**Table 3:** Ingredients (% as-fed basis) of grower experimental test diets fed to YPM × Ross 708 male broilers from 12 to 18 d of age (experiment 2).

Ingredient, %	Test Diet USA	Test Diet ARG	Test Diet BRA
Corn USA	47.25	-	-
Corn ARG	-	47.25	-
Corn BRA	-	-	47.25
Dextrose	47.25	47.25	47.25
Limestone	1.27	1.27	1.27
Dicalcium phosphate, 18% P	2.08	2.08	2.08
Salt	0.20	0.20	0.20
Sodium bicarbonate	0.20	0.20	0.20
Choline chloride, 60%	0.25	0.25	0.25
Trace mineral premix ^1^	0.50	0.50	0.50
Vitamin premix ^2^	0.50	0.50	0.50
Titanium dioxide	0.50	0.50	0.50

^1^ Mineral premix includes per kg of diet: Mn (manganese sulfate), 120 mg; Zn (zinc sulfate), 100 mg; Fe (iron sulfate monohydrate), 30 mg; Cu (tri-basic Cu chloride), 8 mg; I (ethylenediaminedihydroxide), 1.4 mg; Se (sodium selenite), 0.3 mg. ^2^ Vitamin premix includes per kg of diet: Vitamin A (Vitamin A acetate), 187,390 IU; Vitamin D (cholecalciferol), 6614 IU; Vitamin E (DL-alpha tocopherol acetate), 66 IU; menadione (menadione sodium bisulfate complex), 4 mg; Vitamin B12 (cyanocobalamin), 0.03 mg; folacin (folic acid), 2.6 mg: D-pantothenic acid (calcium pantothenate), 31 mg; riboflavin (riboflavin), 22 mg; niacin (niacinamide), 88 mg; thiamin (thiamin mononitrate), 5.5 mg; D-biotin (biotin), 0.18 mg; and pyridoxine (pyridoxine hydrochloride), 7.7 mg.

**Table 4:** Gross energy and apparent metabolizable energy (kcal/kg dry matter) of corn from different origin fed to YPM × Ross 708 male broilers from 12 to 18 d of age (experiment 1).

Treatment	Gross Energy	Apparent Metabolizable Energy	
	kcal/kg	kcal/kg ^1^	% of Gross Energy
**USA**	4417	3651	82.66
**ARG**	4422	3666	82.90
**BRA**	4396	3694	84.04
**SEM ^2^**	-	21.60	-
***p*** **-value**	-	0.370	-

^1^ Least-square means of 12 replicate pens, with 9 birds each. ^2^ Standard error of the mean.

**Table 5:** Apparent ileal amino acid digestibility (%) of corn of different origin fed to YPM × Ross 708 male broilers from 12 to 18 d of age (experiment 2).

**Treatment**	**Apparent Ileal Digestibility ^1^**					
	**Met**	**Cys**	**Met + Cys**	**Lys**	**Thr**	**Val**
USA	85.00	71.94	77.91	68.11	64.08	74.44
ARG	84.60	73.38	77.82	68.56	66.66	75.72
BRA	86.74	75.09	80.58	70.44	66.75	75.71
SEM ^2^	0.95	1.23	1.09	2.17	1.56	1.38
*p*-value	0.242	0.184	0.131	0.713	0.400	0.754
**Treatment**	**Apparent Ileal Digestibility ^1^**					
	**Ile**	**Trp**	**Leu**	**Phe**	**His**	**Arg**
USA	78.71	72.53 ^b^	87.51	82.50	82.92	78.90
ARG	79.81	73.30 ^ab^	88.09	83.42	83.19	78.77
BRA	79.90	77.45 ^a^	87.94	83.52	83.21	81.17
SEM ^2^	1.16	1.32	0.68	0.93	0.80	1.37
*p*-value	0.722	0.024	0.829	0.694	0.961	0.376

^a,b^ Least square means within a column with different superscripts differ significantly (*p* ≤ 0.05). ^1^ Least-square means of 12 replicate pens, with 9 birds each. ^2^ Standard error of the mean.

## Data Availability

The data presented in this study are available from the corresponding authors upon reasonable request.
